# Statins and the incidence of post-stroke depression: a systematic review and meta-analysis

**DOI:** 10.3389/fneur.2024.1486367

**Published:** 2025-01-07

**Authors:** Chaohua Cui, Jue Li, Weicong Chen

**Affiliations:** Life Science and Clinical Medicine Research Center, Affiliated Hospital of Youjiang Medical University for Nationalities, Baise, Guangxi, China

**Keywords:** post-stroke depression, statins, incidence, stage and region, type and dose

## Abstract

**Introduction:**

Post-stroke depression (PSD) can lead to poorer functional outcomes and prognosis. Brain inflammation is a risk factor for PSD. Statins might be beneficial due to their anti-inflammatory properties. Different studies have yielded varying results regarding the effects of statins. Therefore, this meta-analysis aims to clarify the effect of statins on PSD.

**Methods:**

Objectives: To evaluate the relationship between PSD and the use of statins. Data Sources: Databases including PubMed, Web of Science, Embase, and Cochrane Library. Eligibility Criteria: Original observational cohort studies. Participants: Patients with ischemic stroke. Interventions: Use of statins. Appraisal and Synthesis Methods: Forest plot to display pooled results; *I*^2^ test to evaluate heterogeneity.

**Results:**

Of the 37 studies selected, four were eligible. The four studies included 93,893 patients (with statins: 45,598) and more than 17,470 PSD patients. The mean age ranged from 62.1 to 70.8 years. The percentage of female participants ranged from 42.1% to 57.9%. For PSD in different regions, the pooled OR for all regions using random-effects methods was 1.21 (95% CI: 0.44–3.33). The pooled OR for Asian populations was 1.42 (95% CI: 0.37–5.40), and for European populations, it was 0.76 (95% CI: 0.73–0.78). The pooled OR for all regions using fixed-effects methods was 0.84 (95% CI: 0.81–0.86). The pooled OR for Asian populations was 2.62 (95% CI: 2.34–2.93).

**Conclusion:**

Depending on the pooling method used, statin use in Asia either increased or had no relationship with PSD. For European patients, statin use reduced the incidence of PSD.

## Introduction

Post-stroke depression (PSD) is a common complication for stroke patients (1). Stroke patients have an eight times higher incidence of depression than the general population (2, 3). PSD can lead to poorer functional outcomes, prognosis, and cognitive impairment (2). These can further lead to a reduced quality of life and increased mortality. Although antidepressants are a standard treatment for depression, the longer treatment cycle and adverse effects result in poor compliance among some patients (2). Therefore, several studies have investigated novel drugs, such as statins, known for their anti-inflammatory properties, for the treatment of PSD (3–9).

Some studies have found that brain inflammation is a risk factor for PSD (3). Therefore, anti-inflammation has become a new target for PSD treatment. Statins exhibit anti-inflammatory effects on the brain by reducing levels of IL-6 and IL-8 (3–6). Consequently, statins have emerged as a candidate drug for post-stroke depression (PSD) due to their anti-inflammatory and neuroprotective properties (3, 4). Some studies have explored the relationship between statins and PSD in clinical cohorts (3, 5–9). However, it is puzzling that these studies have produced distinct results regarding this relationship. Choi et al.'s study concluded that statins have no effect on the incidence of PSD (5). Studies by Kang et al. and Kim et al. both found that statins could decrease the incidence of PSD (6, 7). Conversely, Kang et al.'s study indicated that statins could increase the incidence of PSD (8). Li et al.'s study suggested that pre-stroke use of statins could decrease the incidence of PSD, but continued use post-stroke might increase the incidence (9). One large cohort study in Denmark suggested that statins reduced the incidence of PSD (3). These complex conclusions make it difficult to evaluate whether PSD patients could benefit from statins. The differing regions of the aforementioned studies, with varying demographic, environmental, or genetic factors, could partly explain the divergent results. Asian and Danish populations exhibited different reactions to statins due to varying baseline low-density lipoprotein levels and metabolic differences (10). Additionally, lower cholesterol levels, which could cause PSD by altering neurotransmitters and the hypothalamus-pituitary axis, might offset the anti-inflammatory effects of statins (5, 10). Furthermore, statin use's type, dose, and duration could also have differential effects on PSD (6, 9).

Statins are a common long-term treatment for stroke patients. As a standard treatment for stroke prevention, statins are necessary to clarify the relationship between statins and PSD. Therefore, we conducted this systematic review and meta-analysis to explore whether statins affect the incidence of PSD and whether this relationship is affected by different regions, doses, or types of statins.

## Methods

The meta-analysis was designed according to the MOOSE (Meta-analysis Of Observational Studies in Epidemiology) guidelines. Eligible study articles were screened and evaluated, data were collected and analyzed, and results were reported.

### Search strategy

We searched PubMed, Web of Science, Embase, and Cochrane Library. First, we selected articles published before July 31, 2024. We used the keywords [(“statin”/exp OR statins) AND depression AND (“stroke”/exp OR “ischemic stroke” OR (“cerebrovascular disease”/exp)] for our search. We then obtained articles to evaluate statin use's effect on PSD incidence.

### Inclusion and exclusion criteria

The inclusion criteria were: (1) patients with ischemic stroke; (2) intervention involving the use of statins; (3) cohort study design; and (4) outcomes including the incidence of post-stroke depression. The outcomes include the value of ORs.

The exclusion criteria were: (1) Studies lacked sufficient sample power when involving fewer than 50 patients; (2) Studies that did not report outcomes as odds ratios (ORs) or lacked sufficient data to calculate ORs were excluded; (3) animal studies; and (4) case reports, letters, comments, meeting reports, and unpublished studies.

### Study selection

We (Chaohua Cui, Jue Li, and Weicong Chen) independently screened the eligible studies. We initially selected articles after reading their titles and abstracts. Then, after reading the full texts, we further excluded articles that did not have suitable outcome data. We further supplemented qualitative studies using references from these articles and related review articles. We did not exclude studies based on language or geographic location. Disagreements regarding the same study were resolved through discussion.

### Data collection and quality evaluation

We (Jue Li and Weicong Chen) independently extracted the data, including the name of the author, date of publication, name of the journal, region of the study, number of subjects, age of subjects, study design, timing of statin use, duration of statin use, follow-up time, and values of the ORs and 95% CIs. The regions of the study were divided into Asia and Europe. The study designs were divided into prospective and retrospective cohorts. The timing of statin use was divided into pre-onset and post-onset. The duration of statin use was divided into 1 month and 1 year. The follow-up times were divided into < than 1 year, 1 year, and more than 1 year.

Two authors (Chaohua Cui and Jue Li) independently evaluated the quality of the studies using the Newcastle–Ottawa Scale. The scale ranges from 1 to 9 stars. Studies with more than five stars were considered to be of good quality. A high-quality article indicates better accuracy of intervention factors, better representativeness of the cohort, more suitable comparability of the control, better baseline balance, accuracy in evaluating PSD, sufficient follow-up time, and integrity and rationale for patients lost to follow-up. Disagreements regarding the same study were resolved through discussion.

### Statistical analysis

We pooled the OR and 95% CI values using forest plots and conducted subgroup analyses by region (Asia, Europe). Heterogeneity was assessed using the *I*^2^ test. If the *I*^2^ value was ≥50%, the data were considered to exhibit significant heterogeneity based on previous research (11). The Der Simonian-Laird random-effects method was employed to pool the data, given the anticipated heterogeneity across observational studies. Publication bias was assessed using Egger's test; *p* < 0.05 indicated significant publication bias, and the results were displayed with Egger's publication bias plot. Additionally, sensitivity analysis was performed using the trim and fill method, and the results were displayed using a funnel plot. All data were processed using the statistical software Stata/MP 14 (Stata Corp, College Station, TX, USA).

## Results

### Study identification and description

We identified 37 articles from the databases and downloaded 11 of them. Ultimately, our meta-analysis included four eligible studies (Supplementary Figure 1). These four studies included 93,893 patients (45,598 with statins and 48,295 without statins). Three of the studies included 17,470 patients with post-stroke depression. The mean age of the patients ranged from 62.1 to 70.8 years. The percentage of female participants ranged from 42.1% to 57.9%. Three studies were conducted in Asia and one in Europe. All included studies were of good quality (Table 1 and Supplementary Table 1).

**Table 1:** Baseline characteristics of patients in the included studies.

**References**	**Age**	**Female (%)**	**Region**	**Study design**	**Follow-up duration**
Wium-Andersen et al. (3)	N/A	47.6	Europe	Retrospective	1 year
Kim et al. (7)	64.5	42.3	Asian	Prospective	1 year
Kang et al. (8)	70.8	57.9	Asian	Retrospective	1 year
Li et al. (9)	62.1	42.1	Asian	Retrospective	1 year

### Statin use and PSD risk

**Figure 1:**
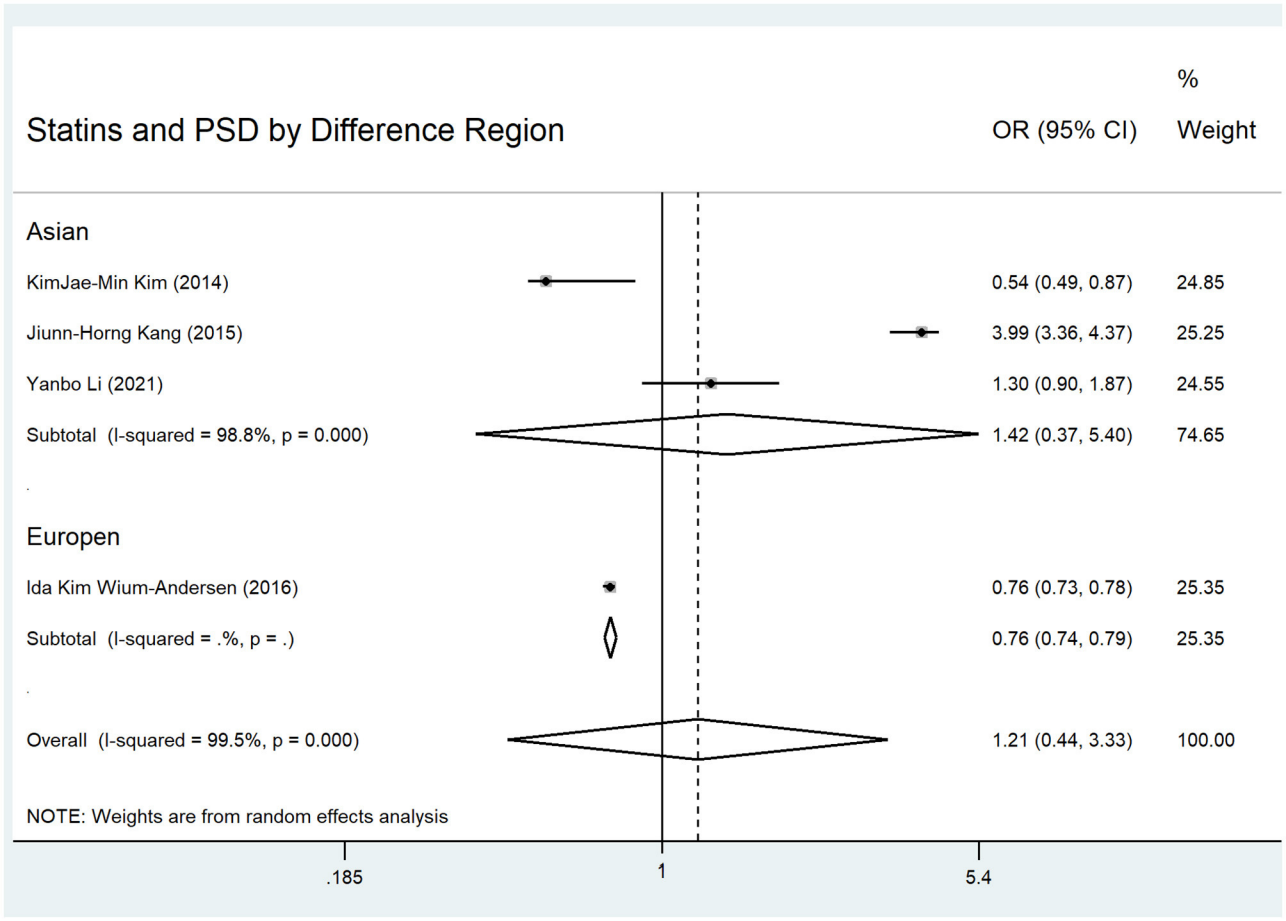
Forest plots for statins and PSD in different regions using random methods.

**Figure 2:**
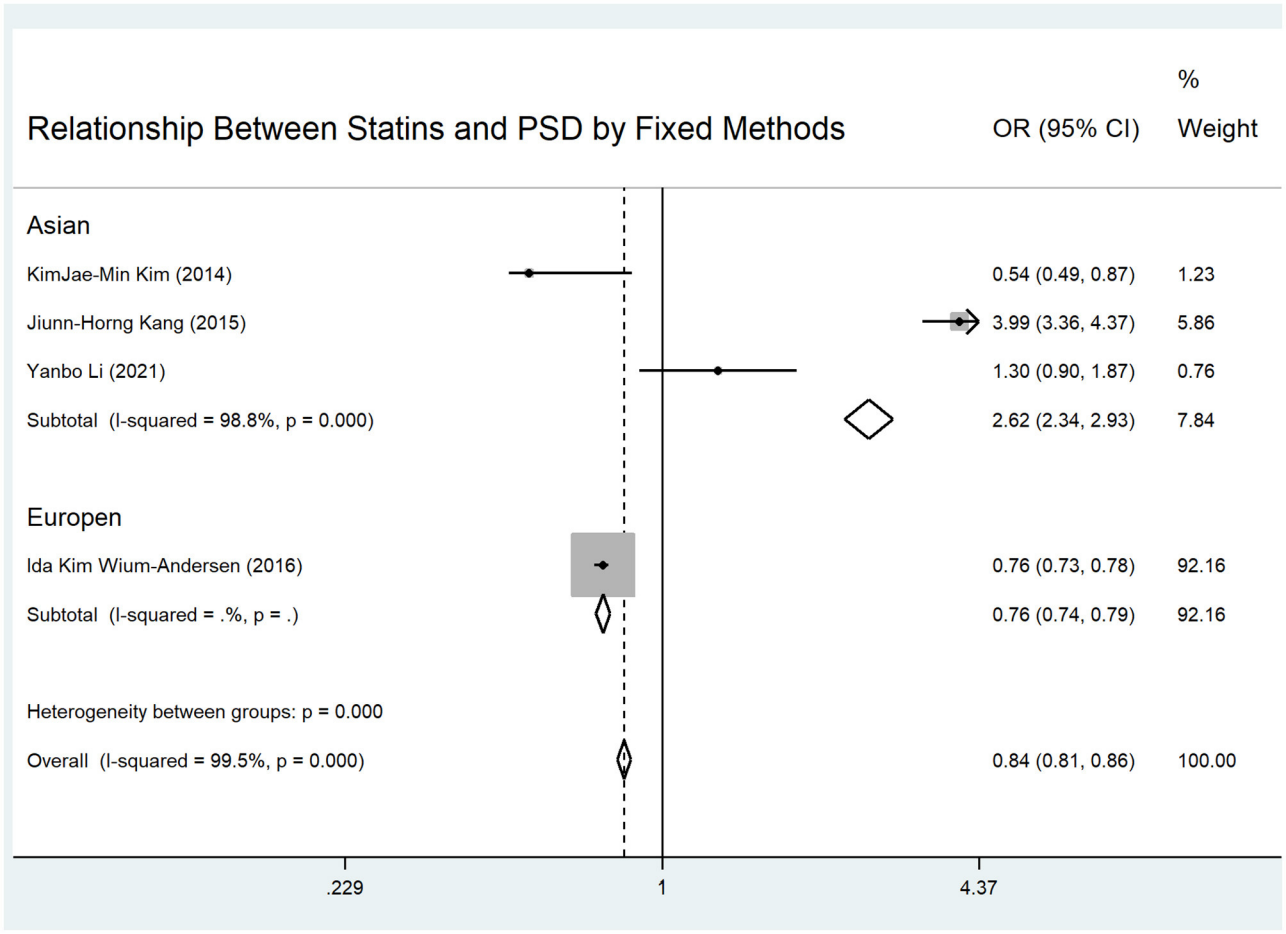
Forest plots for statins and PSD in different regions using fixed methods.

In examining statin use and PSD incidence within the first year, the pooled OR of the four eligible studies (3, 7–9) with random-effects methods was 1.21 (95% CI: 0.44–3.33), which was not statistically significant. The *I*^2^ was 99.5%, indicating significant heterogeneity among the studies (Figure 1). In the regional subgroup analysis, the OR for the Asian subgroup (7–9) was 1.42 (95% CI: 0.37–5.40), which was not statistically significant. The *I*^2^ was 98.8%, indicating significant heterogeneity (Figure 1). The pooled OR of the four eligible studies (3, 7–9) with fixed-effects methods was 0.84 (95% CI: 0.81–0.86), which was statistically significant. The *I*^2^ was 99.5%, indicating significant heterogeneity (Figure 2). In the regional subgroup analysis with fixed-effects methods, the OR for the Asian subgroup (7–9) was 2.62 (95% CI: 2.34–2.93), which was statistically significant. The *I*^2^ was 98.8%, indicating significant heterogeneity (Figure 2). The results of fixed-effects methods differed from those of random-effects methods because the former do not account for heterogeneity between studies. The OR for the Europe subgroup was 0.76 (95% CI: 0.73–0.78), which was statistically significant.

**Figure 3:**
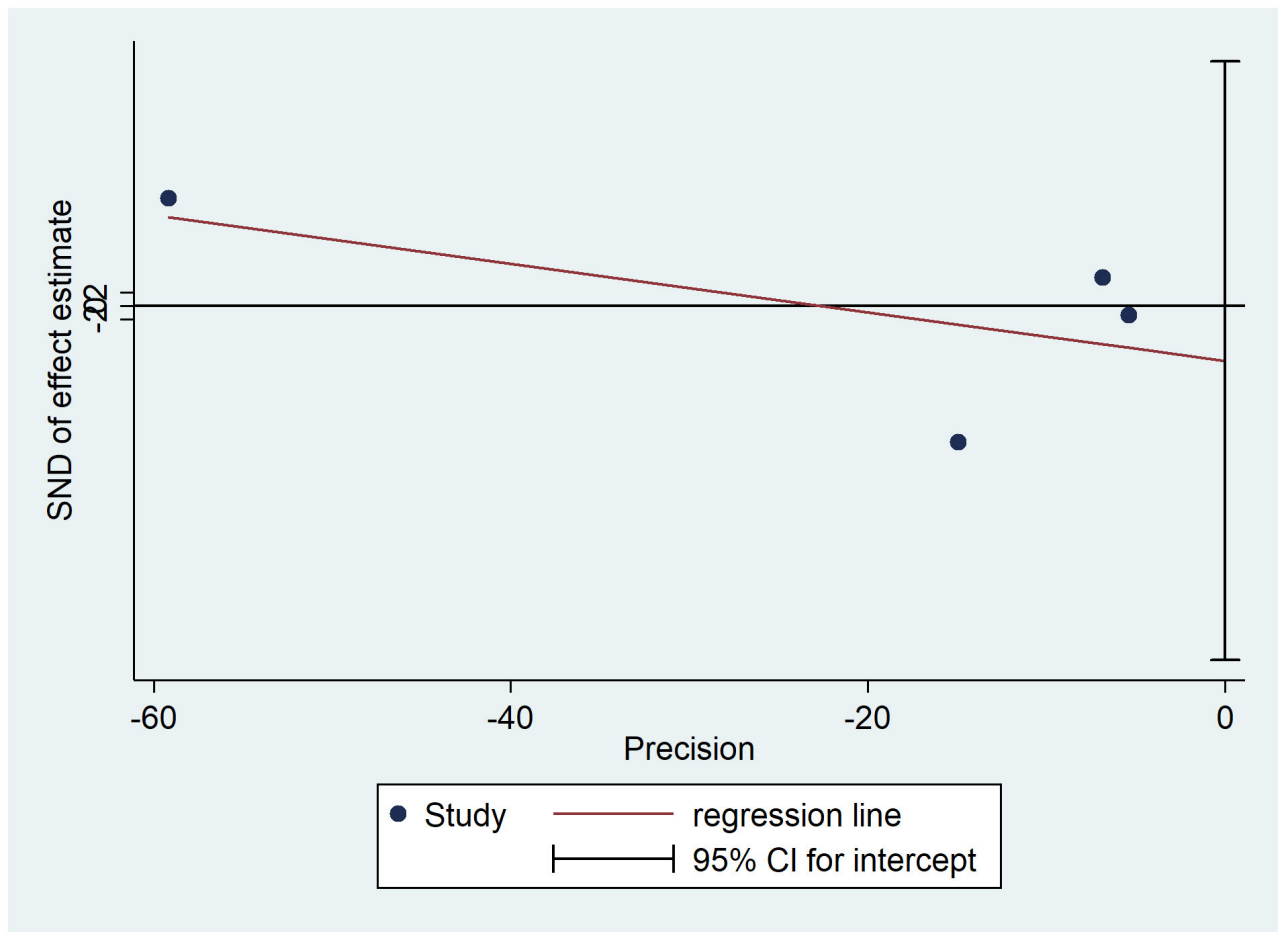
Egger's publication bias plot for statins and PSD.

All pooled studies' publication bias and sensitivity analysis results are shown as funnel plots in Figure 3 and Supplementary Figure 2. The study did not show significant publication bias (Figure 3). The test of H0 showed no small-study effects (*P* = 0.513). The sensitivity analysis did not alter the statistical significance for the 95% CI (Supplementary Figure 2). The test for heterogeneity showed Q = 589.681 with 3 degrees of freedom (*p* < 0.001), indicating significant heterogeneity between the studies. After the trim process, the pooled OR using fixed-effects methods was 0.757 (95% CI: 0.734–0.780), and the pooled OR using random-effects methods was 0.716 (95% CI: 0.291–1.761).

### Differences in statin use duration and PSD risk

One study by Kim et al. found a reduction in PSD within the first year after onset, but it did not affect PSD at baseline (2 weeks after onset) 7. Another study by Wium-Andersen et al. demonstrated that statins reduced PSD within both 1 and 10 years (3). However, another study suggested that statins did not affect PSD between 6 and 64 months (5). Another study indicated that using statins both before and after onset could increase PSD, while using them only before onset could decrease PSD (9). An additional study indicates that statin-associated PSD may be dose-sensitive or time-dependent in stroke patients (8). Overall, the findings suggest that both the duration and timing of statin use may influence PSD outcomes, with effects varying across studies.

## Discussion

Our meta-analysis identified a complex relationship between statin use and PSD, highlighting notable differences between Asian and European populations, which may be influenced by dosage, duration, and metabolic factors. The pooled data using random-effects methods showed that statin use was unrelated to PSD. In the regional subgroup analysis using random-effects methods, statin use was not related to PSD in Asian patients but reduced PSD in European patients. These studies did not exhibit significant publication bias. Significant heterogeneity (*I*^2^ > 99%) was observed between studies, which could stem from differences in populations, statin types, or study designs. However, the pooled results were credible, as sensitivity analyses did not alter the statistical significance. The duration of statin use and PSD onset timing could have distinct effects.

The pooled results using random-effects methods showed that statins did not increase the incidence of PSD. However, the pooled results using fixed-effects methods showed that statins reduced the incidence of PSD. The differing results of the three Asian studies influenced the overall conclusion (7–9). Additionally, the pooled results of the Asian studies affected the overall regional results. A single European study with a large population showed that statins reduced the incidence of PSD (3). However, this study did not receive sufficient weight in the random-effects analysis (25.35%). In the fixed-effects analysis, this study received a significant weight (92.16%). The other Asian studies had similar weight changes between fixed- and random-effects methods. Therefore, the pooled results of fixed- and random-effects methods yielded distinct outcomes. Although the fixed-effects method might not be suitable for the meta-analysis as it does not account for heterogeneity between studies, given the significant population differences between studies, the weighting of the four studies in the fixed-effects analysis appears more reasonable. Even though these studies had significant heterogeneity, we cannot ignore the pooled results from the fixed-effects analysis. The European study with a large population deserved the highest weight, particularly in the pooled results.

The pooled results for the Asian subgroup presented a more complex scenario. Kang et al.'s study, which included more patients, significantly impacted the pooled results for Asians (8). Therefore, the pooled results using fixed-effects methods were similar to Kang's. Interestingly, statins had utterly opposite effects on PSD between Asian and European patients.

The differing metabolic characteristics of statins, variations in cholesterol levels, and population differences between Asian and European patients may partly explain these results (10). Asian patients have been observed to be more sensitive to statins (10). Statins may affect PSD by influencing cholesterol levels, which subsequently impact brain function. According to our previous and other studies, lower doses of statins may achieve better effects in Asian stroke patients (11–14). Therefore, Asian patients taking statins could have lower cholesterol levels. Lower cholesterol levels could cause depression in stroke patients (4, 5). However, the critical point is whether the influence of serum cholesterol on the CNS reaches a threshold that causes functional impairment, which could then lead to depression (5). Therefore, the extent to which statins reduce serum cholesterol could affect the association between statin use and PSD.

The average LDL-C levels in Western societies significantly exceed those in Asian societies (5). Consequently, statins may reduce serum cholesterol levels more effectively in Asians compared to Europeans and Americans. This may also account for the varying relationships between statins and PSD across different regions. Based on these findings, low-dose statins could represent a practical approach for Asian patients. Due to their reduced effect on serum cholesterol levels in Asian patients, low-dose statins might have a diminished impact on post-stroke depression.

The multifaceted effects of statins may also be attributed to their anti-inflammatory properties (15). Inflammation in the brain is a key mechanism underlying depression (3). Statins exert immune-modulating and anti-inflammatory effects on the central nervous system (15). Statins may alleviate depression through their anti-inflammatory effects in the brain (3, 7). However, for patients with PSD, the anti-inflammatory effects are not unequivocally beneficial. One study found that statins reduced the risk of both early-onset and late-onset PSD (3). Other studies have identified distinct roles of inflammatory reactions during the acute and chronic stages of ischemic stroke (15, 16). Furthermore, studies have observed varying effects of statins at different stages of stroke (6, 9). These findings suggest that the use of statins may necessitate different strategies and approaches at various stages of PSD in patients. The specifics of these strategies and approaches require further investigation.

This study has several limitations. First, our meta-analysis included four prospective and retrospective observational studies. The data were aggregated from various types of studies that differed in design and execution, leading to substantial heterogeneity. However, sensitivity analysis indicated that the studies did not alter the pooled results. Therefore, the pooled results are credible. Randomized controlled trials are needed to further validate the results. Secondly, the majority of studies originate from Asia. Studies from other regions are required to balance the current Asia-centric data pool. Thirdly, statins may have varying effects at different stages post-stroke. Therefore, further exploration of the relationship between statins and PSD could provide actionable insights for clinicians managing PSD at various stages of stroke recovery. Differences in stroke type and location could also result in varying effects of statins on patients (17, 18), warranting further study.

## Conclusion

In conclusion, this analysis indicates that statins may provide distinct benefits related to PSD, contingent upon patient population and treatment specifics. Clinicians may consider dose adjustments and population-specific guidelines to optimize PSD management; however, further research is necessary to substantiate these findings.

## Data Availability

The original contributions presented in the study are included in the article/Supplementary material, further inquiries can be directed to the corresponding author/s.
