# Supplementary material for: Statins and the incidence of post-stroke depression: a systematic review and meta-analysis
Source: Front Neurol. 2025 Jan 7;15:1486367. doi: 10.3389/fneur.2024.1486367 (PMC11746108; doi:10.3389/fneur.2024.1486367)
Supplement: Supplementary file 1 [file Table_1.docx]

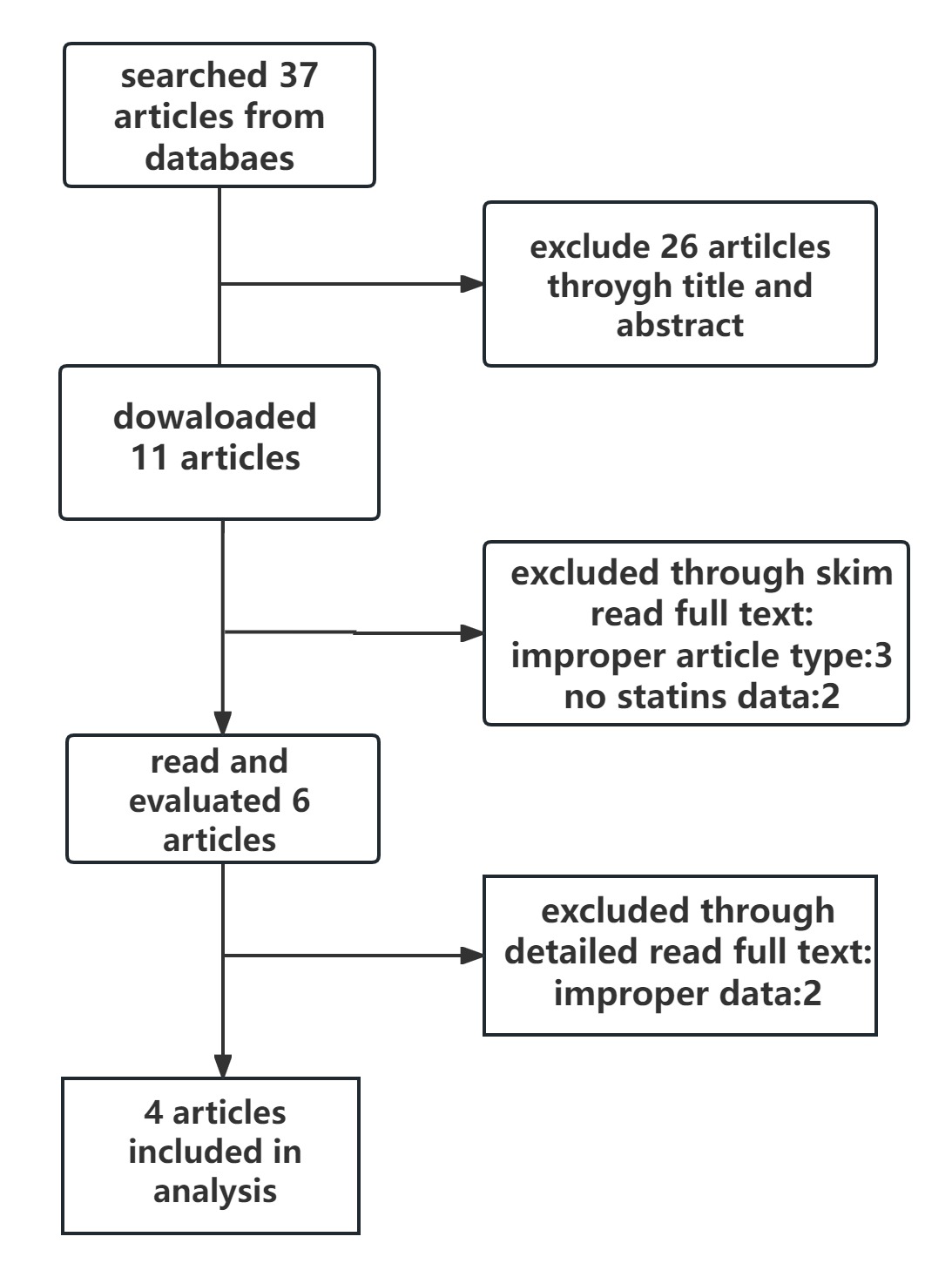


Supplementary Figure 1: Flowchart of included studies


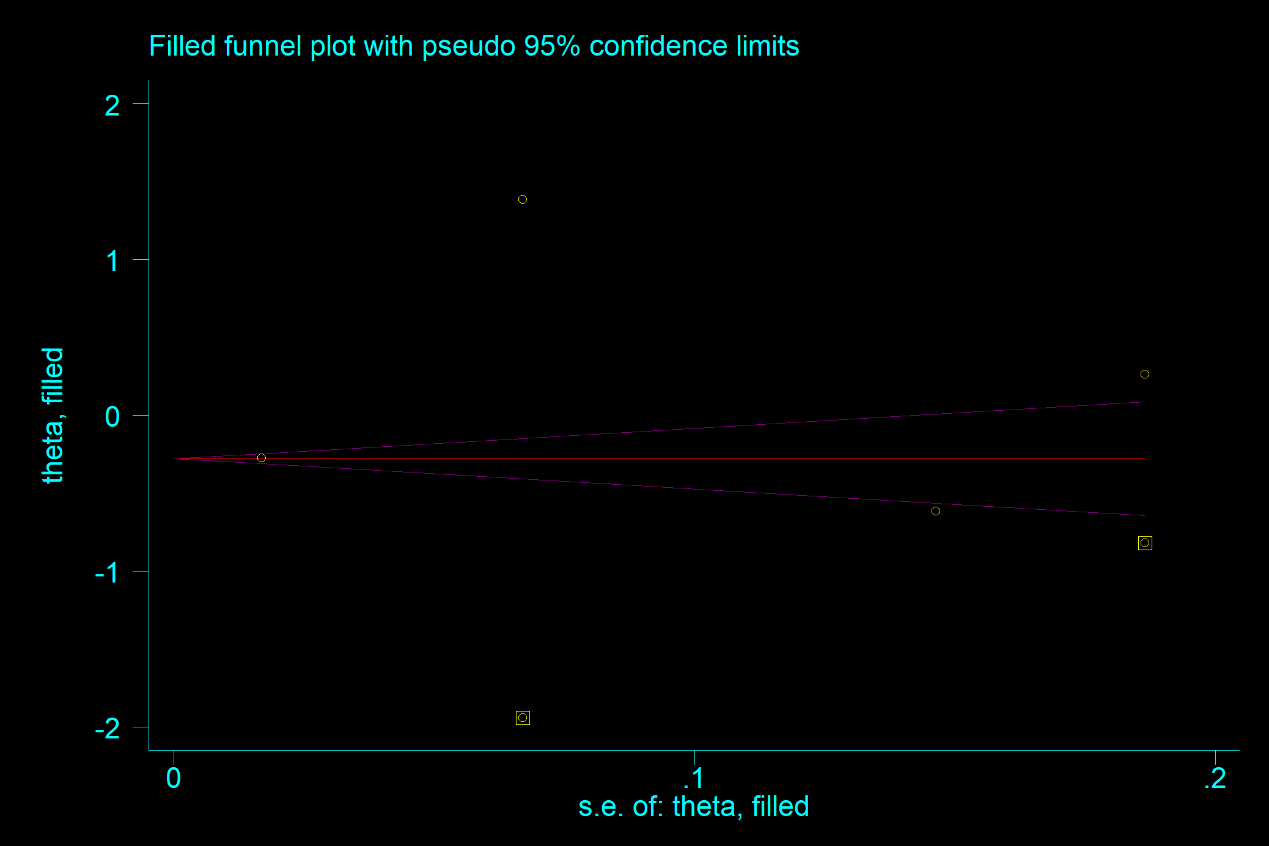


Supplementary Figure 2: Funnel Plots for Trim and Fill Methods: Statins and PSD

| **Author** | **Publish**  **Year** | **Number of Totals** | **Number of Statins** | **Number of PSD** | **Quality** |  |
| --- | --- | --- | --- | --- | --- | --- |
| Ida Kim Wium-Andersen [03] | 2016 | 80681 | 41307 | 17185 | 7* |  |
| Jae-Min Kim [7] | | 2014 | 423 | 251 | 75 | 7* |
| Jiunn-Horng Kang [8] | 2015 | 11218 | 2847 | N/A | 5* |  |
| Yanbo Li [9] | 2021 | 1571 | 1193 | 210 | 6* |  |

Supplementary Table 1 Other Characteristics of Patients in the Included Studies
